# Bacteriocin synthesis in uropathogenic and commensal *Escherichia coli*: colicin E1 is a potential virulence factor

**DOI:** 10.1186/1471-2180-10-288

**Published:** 2010-11-15

**Authors:** David Šmajs, Lenka Micenková, Jan Šmarda, Martin Vrba, Alena Ševčíková, Zuzana Vališová, Vladana Woznicová

**Affiliations:** 1 Department of Biology, Faculty of Medicine, Masaryk University, Kamenice 5, Building A6, 625 00 Brno, Czech Republic; 2 Department of Clinical Microbiology, Faculty Hospital Brno, Jihlavská 20, 625 00 Brno, Czech Republic; 3 Department of Medical Microbiology, Faculty of Medicine, Masaryk University, Pekařská 53, 656 91 Brno, Czech Republic

## Abstract

**Background:**

Bacteriocin production is an important characteristic of *E. coli *strains of human origin. To date, 26 colicin and 9 microcin types have been analyzed on a molecular level allowing molecular detection of the corresponding genes. The production incidence of 29 bacteriocin types and *E. coli *phylogroups were tested in a set of 361 *E. coli *strains isolated from human urinary tract infections (UTI) and in 411 control strains isolated from feces of patients without bacterial gut infection.

**Results:**

Production of 17 and 20 individual bacteriocin types was found in the UTI and control strains, respectively. Microcin H47 encoding determinants were found more often among UTI strains compared to controls (37.9% and 27.0% respectively, p = 0.02) and strains producing microcin H47 belonged predominantly to phylogroup B2 when compared to other bacteriocin producers (67.4% and 36.7%, respectively; p < 0.0001). Producers of 3 or more identified bacteriocin types were more common in the UTI group (20.0% compared to 12.4% in controls, p = 0.03). In the UTI strains, there was a markedly higher number of those producing colicin E1 compared to controls (22.1% to 10.2%, respectively, p = 0.0008). Moreover, colicin E1 production was more common in the UTI bacteriocinogenic strains with multi-producer capabilities. As shown by Southern blotting, pColE1 DNA was not recognized by the ColIa probe and *vice versa *suggesting that pColE1 was independently associated with pColIa in UTI strains.

**Conclusion:**

*E. coli *strains isolated from human urinary tract infections showed increased incidence of microcin H47 and colicin E1 production, respectively. Moreover, colicin E1 itself appears to be a potentially important virulence factor of certain uropathogenic *E. coli *strains.

## Background

Bacteriocins are a diverse group of gene encoded antibacterial agents including corpuscular bacteriocins, colicins, and microcins. While there is indirect evidence of presence of corpuscular bacteriocins in the genus *Escherichia *[1], they have not been unequivocally identified in this genus where only production of proteinaceous colicins and low molecular weight microcins has been directly demonstrated. Both colicins and microcins have a relatively narrow spectrum of activity, predominantly comprising strains of the same species (colicins) and strains of the same and related species (microcins).

Uropathogenic strains of *E. coli *(UPEC) form a subgroup of extra-intestinal pathogenic *E. coli *(ExPEC) strains and cause human urinary tract infections (UTI). Previous studies showed that there are several virulence factors associated with UPEC strains including adhesins, α-hemolysin and aerobactin production, cytotoxic necrotizing factor, and microcin V (previously known as colicin V) [2-7]. The ColV plasmids (i.e. in present terminology microcin V encoding plasmids) have been found to be associated with increased pathogenicity of *E. coli *strains [8]. The microcin V encoding gene, *cva*C, has been found more frequently in cases of pyelonephritis compared to cases of other clinically distinct UTI infection syndromes, including cystitis and prostatitis [9], suggesting a possible role for the genes located on the microcin V-encoding plasmids in the pathogenesis of pyelonephritis. Moreover, bacteremic isolates of *E. coli *strains were more often characterized by plasmid encoded microcin V production [10] whereas in intestinal strains, microcin V was most often chromosomally encoded. Nevertheless, there are contradictory results regarding the role of microcin V in bacterial virulence [11,12].

Bacteriocin production is an important characteristic of *E. coli *and several related species in the *Enterobacteriaceae *family. Within the genus *Escherichia*, bacteriocin production is almost exclusively associated with strains of *E. coli *[13]. Moreover, there is increasing evidence indicating that bacteriocins are important elements in bacterial ecology and are linked to their possible probiotic effects [14-18]. However, the precise ecological role of bacteriocins in microbial competitions among different bacterial populations in complex bacterial communities is not yet exactly known. The variability of bacteriocin types, different modes of molecular action, varying entry routes into susceptible bacteria, and the number of additional genes present on bacteriocin genophores are just some of the obfuscating factors. To date, 26 colicin types [19-22] have been described in detail. In addition, nine microcin types have been analyzed on a molecular level allowing molecular detection of the corresponding genes [23-25]. To study the clinical and ecological importance of bacteriocin synthesis, detection of their specific types is needed due to their diversity and also due to the molecular diversity of the genes encoded on the plasmids or chromosomal regions in question. In this communication, we compare colicin and microcin types identified in two groups of *E. coli *strains isolated from healthy human guts and from human urinary tract infections.

## Results

### Detection system for 23 different colicin types

Primers shown in Additional file 1 were used to detect 23 colicin types and microcin C7. The detection system for 5 additional microcin types including mB17, mH47, mJ25, mL, and mV was taken from Gordon and O'Brien [26]. With the exception of cloacin DF13, pesticin I, and bacteriocin 28b, this system is able to detect all colicin types so far characterized on a molecular level. All primer pairs were tested on all 23 established colicin type producers to detect cross-reactivity with other colicin types. Cross-reactivity of the PCR amplification tests was observed in the following combinations: primers for colicin E3 gene also detected colicin E6; E6 primers also detected colicins E2, E3, E5, E8 and E9; E7 primers also detected colicin E4; E8 primers also detected colicin E7; Ib primers also detected colicin Ia; colicin U primers also detected colicin Y and *vice versa *and primers for colicin 5 also detected colicin 10. Identification of cross-reacting colicin producers therefore required sequencing of the corresponding amplicons, which was performed for all identified colicins E2-E9, Ia-Ib, U-Y, and 5-10.

### Bacteriocin mono- and multi-producers among the control and UTI strains

Bacteriocin types identified in control and UTI strains are shown in Table 1 and statistically significant differences between bacteriocin producing and non-producing strains are shown in Table 2. In the UTI *E. coli *strains, 195 bacteriocin producing strains (54.0%) were identified among 361 tested. This incidence was not significantly different from bacteriocin producers in the control strains (226 out of 411, 55.0%). Mono-producers and strains producing two identifiable bacteriocin types (double producers) were similarly distributed among both UTI and control groups (mono-producers: 48.7% and 45.6%, respectively; double producers: 30.1% and 28.2%, respectively). Within bacteriocin mono-producers, reduced frequency of strains producing either colicin Ia or Ib was found (5.1% and 13.7% among UTI strains and controls, respectively, p = 0.003). Bacterial strains with 3 or more bacteriocin encoding determinants were significantly more common in the UTI group (20.0% compared to 12.4% in controls, p = 0.03). Both UTI and control strains showed a similar percentage of unidentified bacteriocin types (6.2% and 8.8%, respectively), indicating the presence of, as yet, unknown bacteriocin versions or types in *E. coli *strains.

**Table 1:** List of control and UTI *E. coli *strains producing bacteriocins and identified colicin and microcin types

Control *E. coli *strains			UTI *E. coli *strains		
Identified bacteriocin types*	**No. of strains producing specific bacteriocin types or combination thereof**	**Frequency among producer strains in %** **(n = 226)**	**Identified bacteriocin types**	**No. of strains producing specific bacteriocin types or combination thereof**	**Frequency among producer strains in %** **(n = 195)**

micH47	47	20.8	micH47	60	30.8
Ia	22	9.7	Ia, micV	25	12.8
Ia, micV	21	9.3	E1, Ia, micV	10	5.1
Ib	9	4.0	Ia	8	4.1
Js	9	4.0	M	7	3.6
micV	9	4.0	micV	5	2.6
B, M	7	3.1	E1, micV	4	2.1
Ib, micV	6	2.7	E1, M	4	2.1
K	4	1.8	E1	2	1.0
Ia, micH47	4	1.8	Ib	2	1.0
E1, Ia, micV	4	1.8	Js	2	1.0
E1	3	1.3	K	2	1.0
M	3	1.3	E1, Js	2	1.0
E1, Ia	3	1.3	E1, Ia, M	2	1.0
E1, Ib	3	1.3	B, Ia, M	2	1.0
micV, micH47	3	1.3	micV, micH47	2	1.0
micC7	2	0.9	E1, Ia, micH47, micV	2	1.0
E1, K	2	0.9	E2	1	0.5
E1, M	2	0.9	B, M	1	0.5
B, M, micV	2	0.9	E1, Ib	1	0.5
E4, Ia, micV	2	0.9	E1, E2467	1	0.5
Ia, M, micV	2	0.9	E2, micH47	1	0.5
Ib, micH47, micV	2	0.9	E2-9, Ia	1	0.5
E1, Ia, K, micV	2	0.9	E1, micJ25	1	0.5
B	1	0.4	E7, K	1	0.5
E2	1	0.4	E7, micH47	1	0.5
E1, micV	1	0.4	Ia, K	1	0.5
E7, Ib	1	0.4	Ia, M	1	0.5
Ia, Js	1	0.4	Ia, micH47	1	0.5
Ia, K	1	0.4	Ia, Y	1	0.5
Ia, S4	1	0.4	Ib, K	1	0.5
Ia, Y	1	0.4	Ib, micH47	1	0.5
Ia, U	1	0.4	Ib, micV	1	0.5
Ib, M	1	0.4	K, micH47	1	0.5
Js, N	1	0.4	M, N	1	0.5
Js, S4	1	0.4	N, micV	1	0.5
Js, micV	1	0.4	B, E1, M	1	0.5
K, micH47	1	0.4	B, E2, M	1	0.5
N, micH47	1	0.4	B, M, N	1	0.5
N, micV	1	0.4	E1, Ib, micC7	1	0.5
S4, micC7	1	0.4	E1, micC7, micH47	1	0.5
micC7, micH47	1	0.4	Ia, K, micV	1	0.5
micH47, micL	1	0.4	Ia, micC7, micV	1	0.5
B, Ib, M	1	0.4	Ia, N, micV	1	0.5
E1, E4, K	1	0.4	Ib, N, micV	1	0.5
Ia, Js, micV	1	0.4	B, E1, Ib, M	1	0.5
Ia, E2-9, micV	1	0.4	B, E1, M, micV	1	0.5
Ia, K, micV	1	0.4	E1, E2, K, micV	1	0.5
Ia, 5, micV	1	0.4	E1, E3589, Ia, micV	1	0.5
B, Ia, M, micV	1	0.4	E1, Ia, K, micV	1	0.5
B, Ib, M, micV	1	0.4	E1, Js, N, micV	1	0.5
B, M, E2, micV	1	0.4	E1, K, micV, micC7	1	0.5
E1, Ia, M, micV	1	0.4	Ia, K, micH47, micV	1	0.5
E1, Ib, N, micV	1	0.4	B, M, micH47, micV	1	0.5
B, M, N, micV	1	0.4	E1, E7, micH47, micV	1	0.5
B, M, micH47, micV	1	0.4	E1, Ia, micH47, micV	1	0.5
Ia, micC7, micJ25, micV	1	0.4	B, E1, Ia, M, micV	1	0.5
unidentified	20	8.8	E1, E7, Ia, K, micV	1	0.5
			B, E2, K, M, N, micV	1	0.5
			unidentified	12	6.2

*colicin types are given without prefix, mic stands for microcin

**Table 2:** Statistically significant differences in the incidence of bacteriocin encoding determinants among UTI and control *E. coli *strains

Types of bacteriocin producers	No. of UTI bacteriocin producers (%)	No. of control bacteriocin producers (%)	Statistical significance of the difference*
microcin H47 producers	74 (37.9)	61 (27.0)	p = 0.02
producers of colicins Ia and Ib among mono-producers	10 (5.1)	31 (13.7)	p = 0.003
producers of three or more bacteriocins	39 (20.0)	28 (12.4)	p = 0.03
colicin E1 producers	43 (22.1)	23 (10.2)	p = 0.0008
producers of colicin E1 among bacteriocin triple- and multi-producers	28 (14.4)	9 (4.0)	p = 0.0002
bacteriocin E1 and Ia producers	19 (9.7)	10 (4.4)	p = 0.03
bacteriocin E1, Ia, and mV producers	17 (8.7)	7 (3.1)	p = 0.01

*statistical methods derived from the binomial distribution were used (see Methods)

### Bacteriocin types in the control and UTI strains

If the number of individual bacteriocin determinants in each group of producer strains was counted irrespective of the co-produced bacteriocins, 17 and 20 types were identified among the UTI and control strains, respectively. Colicins A, D, E3, E5, E6, E8, E9, 10 and microcin B17 were not detected in either group. In addition to these, colicins E4, S4, U, 5 and microcin L were not detected in the UTI strains.

Among the UTI strains, there was a marked increase in the number of strains producing colicin E1 compared to controls (22.1% to 10.2%, respectively, p = 0.0008). This increased incidence of colicin E1 encoding determinants was not associated with mono-producers or with double producers. However, in triple producers and multi-producers, this association was very strong compared to control strains (14.4% and 4.0% respectively, p = 0.0002). Microcin H47 encoding determinants were found more often among UTI strains compared to controls (37.9% and 27.0% respectively, p = 0.02). Majority of the microcin H47 encoding strains were mono-producers with higher incidence among UTI strains compared to controls (30.8% and 20.8% respectively, p = 0.02).

### *E. coli *phylogroups and colicin production

All investigated strains were phylogenetically analyzed using triplex PCR [27]. There was a marked increase of the B2 genotype in the UTI group compared to controls (59.0% and 42.1%, respectively; p < 0.0001), and a decreased incidence of the A genotype (19.4% and 31.1%, respectively; p = 0.0002). Additionally, a higher incidence of the B2 phylogroup was found in the UTI strains of male origin (74.1%, data not shown) compared with UTI strains of female origin (54.4%, p = 0.001). Distribution of producer and non-producer strains among *E. coli *genotypes is shown in Table 3. In the *E. coli *phylogroup B1, the incidence of bacteriocin producing strains was significantly lower among UTI strains when compared to controls.

**Table 3:** Incidence of bacteriocin producing and non-producing strains among UTI and control strains in *E. coli *phylogroups.

*E. coli *phylogroup A			
	UTI (n = 128)	Control (n = 70)	statistical significance between UTI and control*
Producers	79 (61.7%)	37 (52.9%)	-**
Non-producers	49 (38.3%)	33 (47.1%)	

***E. coli *phylogroup B1**			

	UTI (n = 25)	Control (n = 11)	statistical significance between UTI and control*
Producers	7 (28%)	7 (63.6%)	p = 0.04
Non-producers	18 (72%)	4 (36.4%)	

***E. coli *phylogroup B2**			

	UTI (n = 173)	Control (n = 213)	statistical significance between UTI and control*
Producers	86 (49.7%)	110 (51.6%)	-**
Non-producers	87 (50.3%)	103 (48.4%)	

***E. coli *phylogroup D**			

	UTI (n = 85)	Control (n = 67)	statistical significance between UTI and control*
Producers	54 (63.5%)	41 (61.2%)	-**
Non-producers	31 (36.5%)	26 (38.8%)	

*statistical methods derived from the binomial distribution were used (see Methods, χ^2 ^= 3.41, p = 0.05)

**not statistically significant

The incidence of colicinogenic strains among all strains including both UTI and control strains was higher in the phylogroup A (56.3% and 39.0%, respectively; p = 0.0007) and D (53.4% and 39.0%, respectively; p = 0.009) when compared to B2 (data not shown). In contrast, group B2 showed higher incidence of microcin-encoding strains when compared to strains of group A (56.0% and 37.7%, respectively; p = 0.0003) and D (56.0% and 39.0%, respectively; p = 0.002). Producers of microcin H47 belonged more frequently to group B2 (data not shown) when compared to other bacteriocin producers (67.2% and 36.9%, respectively; p < 0.0001) and less frequently to groups A (10.4% and 35.5%, respectively; p < 0.0001) and B1 (0.7% and 4.5%, respectively; p < 0.04).

### ColE1 plasmids in multi-producer strains

**Figure 1:**
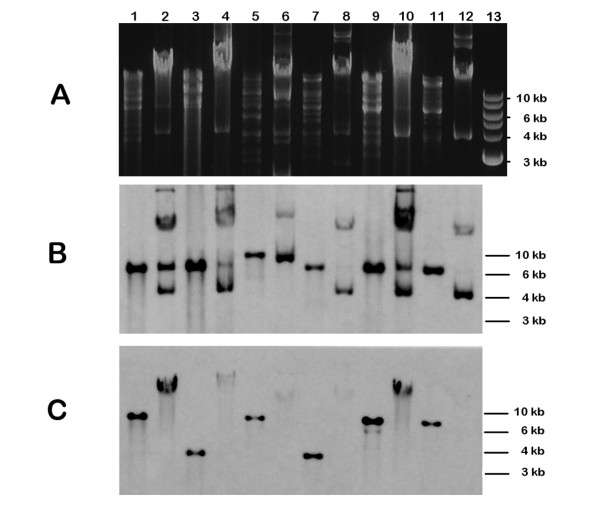
**Detection of DNA encoding colicins E1 and Ia in 6 plasmid DNA samples (out of 12 randomly picked and analyzed colicin E1 producers)**. Panel A: an agarose gel with total plasmid DNA stained with ethidium bromide; panel B: Southern blot analysis of the same plasmid DNA samples with colicin E1 probe; panel C: Southern blot analysis with colicin Ia probe. The 1 kb DNA ladder (New England Biolabs, Ipswitch, MA) was used as the DNA marker (panel A, lane 13). Lanes 1 and 2, plasmid DNA isolated of strain B399 (digested with *Eco*RI and undigested, respectively). Lanes 3 and 4, strain B954 (producer of colicins E1, Ia, and microcin V); lanes 5 and 6, strain B961; lanes 7 and 8, strain B2041; lanes 9 and 10, strain B953; lanes 11 and 12, strain B830. Strains B399, B954, B2041 and B830 were all producers of colicins E1, Ia, and microcin V. Strain B961 produced colicins E1, Ia, E7, K and microcin V. Strain B953 produced colicins E1, Ia, and microcins V and H47. Please note that patterns of undigested plasmid DNA were different in panel B and C, respectively, indicating that colicin Ia and E1 genes are located on separate plasmids.

Strains producing the combination of colicins Ia and E1 were more common in the UTI group compared to controls (9.7% and 4.4%, respectively, p = 0.03) as well as strains producing bacteriocins Ia, E1 and mV (8.7% and 3.1%, respectively, p = 0.01). To test whether these producers synthesizing colicin E1 contain regular low molecular weight pColE1 plasmids or only colicin E1-encoding determinants located on larger plasmids, the plasmid size of 12 colicin E1 producing strains, selected at random, was estimated using the Southern blotting procedure with colicin E1 and colicin Ia probes (Figure 1). Most of these strains synthesized also colicin Ia and microcin V. As shown by Jeziorowski and Gordon [28], when colicin Ia and microcin V co-occur, they are encoded on the same conjugative plasmid as a result of integration of microcin V operon and several other genes into the pColIa plasmid. As shown by Southern blot analysis, all tested colicin E1 producers had low molecular weight plasmid DNA recognized by colicin E1 probe with size ranging from 6.8 to 10 kb (for 6 plasmid samples see Figure 1). To verify the pColE1 plasmid sizes, XL-PCR approach was used to amplify the entire pColE1 using complementary primers recognizing colicin E1 operon. In 10 out 12 samples, a successful DNA amplification resulted in PCR product size ranging between 7.0 - 10 kb (data not shown). The colicin Ia probe hybridized with a higher molecular weight plasmid DNA (Figure 1) indicating that these strains harbor colicin E1 plasmids together with an additional bacteriocin-encoding plasmid.

## Discussion

A detection system for 23 different colicin types was designed and tested. Together with previously published microcin primer set [26], most of the well characterized bacteriocins in the genus *Escherichia *can be identified. Gordon and O'Brien [26] found 102 bacteriocin producing strains among 266 (38%) human *E. coli *strains, whereas in our study, 55% (226/411) of *E. coli *control strains (of similar human origin) were bacteriocin producers. Gordon and O'Brien detected eleven colicin types and seven microcin types. With the exception of microcin M (which co-occurs with microcin H47), all types used in the published study [26] were tested in the present work. Since the identification scheme of bacteriocin producers, including indicator strains and cultivation conditions, differed in both studies, it is likely that the 17% difference reflects the primary identification of producer strains. In our study, 6.2% and 8.8% of strains in both control and UTI strains, respectively, produced unidentified bacteriocins. Appearance of inhibition zones, inducibility with mitomycin C and sensitivity to trypsin suggested that both colicin and microcin types could be expected among untyped producer strains. Some of these strains possibly produce already known, though untested, colicin and microcin types (cloacin DF13, pesticin and bacteriocin 28b, and microcins M, E492, 24, D93). Despite this fact, untyped bacteriocin producers represent an interesting set of *E. coli *strains needing further bacteriocin research.

Both our groups of control strains (taken from two hospitals) were nearly equal in the incidence of bacteriocin types. Since the tributary areas of both hospitals overlap, similarity in incidence of identified bacteriocin types likely reflects the fact that all samples were taken from persons living in the same area of South Moravia, Czech Republic.

No statistically important difference was found in the incidence of bacteriocin producers among UTI strains (54.0% of producer strains) compared to control strains (55.0%). This observation may reflect the fact that most uropathogenic strains originate in the human gut [29]. Investigation of 568 clinical isolates of uropathogenic strains of *E. coli *collected in New Zealand [30] revealed lower incidence of bacteriocin producers (42.6%); an even lower incidence (32.3%) was found among 440 *E. coli *UTI strains tested in 2001 in the Czech Republic [1]. In the latter case, only "rich media" were used for identification of bacteriocin producers, which could result in lower microcin detection in both UTI and control strains (34.8% of control strains were found to be colicinogenic in our study).

Commensal strains of *E. coli *belong mainly to phylogroups A and B1 whereas the group B2 contains highly virulent *E. coli *strains [31]. Virulent *E. coli *strains are also often found in group D. *E. coli *strains in groups B2 and D have the largest genomes [32]. However, there is no exclusive link between *E. coli *groups B2 and D and the ability to cause infection since *E. coli *strains belonging to all groups can cause infection under specific conditions. The observed higher incidence of *E. coli *group B2 among UTI strains, relative to group A, is therefore not surprising. We found that microcin H47 encoding genes are present predominantly in *E. coli *phylogenetic group B2. Since microcin H47 encoding determinants are localized on a bacterial chromosome [33], microcin H47 (and microcin M) genes appears to be often part of genetic elements specific for group B2 [27]. Our findings also suggest that colicin production is principally associated with *E. coli *phylogroup A (and to lesser extent with group D) and not with genotype B2, where microcin producers are more common. As suggested in previous publications [13,34], our results support the model where the colicin producer phenotype, within the *Enterobacteriaceae *family, belongs primarily to commensal intestinal *E. coli *strains.

We found a statistically significant increase in UTI strains producing colicin E1 compared to controls (22.1% and 10.2%, respectively). There was an especially strong association between triple and multiple bacteriocin producers and colicin E1 production - with p-values lower than 0.0005. In a previously published paper [35], ColE1-like plasmids were frequently found among uropathogenic strains of *E. coli *(UPEC). However, no control group was tested to identify the statistical significance of this finding. Among 89 identified bacteriocin producers, 43% were positive for *mob*A-, *rom*- and RNAII-specific sequences [35]; also, since other colicin plasmids may contain the same or highly similar sequences to pColE1 (e.g. pColU) [36], the exact extent of the colicin E1 producing subset is unknown. Based on frequency of incidences of colicin E1 production in our study, the majority of producer strains described by Rijavec et al. [35] containing ColE1-like sequences were probably strains harboring pColE1.

In the group of UTI strains, lower bacteriocin diversity and an increased number of triple and multiple producers were identified. The bacteriocin multi-producer phenotype of UTI strains was predicted as one possible explanation of unidentified colicin types in a previous study [30]. In general, the multi-producer phenotypes require: (i) efficient genetic transfer within the bacterial community, (ii) low habitat heterogeneity to ensure effective negative selection of sensitive bacteria, and (iii) relatively low bacteriocin biosynthesis costs. As experimentally shown by Braude and Siemienski [37] in rats, the first two conditions apply to urinary tract infections suggesting that the multi-producer phenotype is favored among UPEC strains. Besides reduced habitat heterogeneity of the urinary tract compared to the human colon, the multi-producer strains could be more frequently found in UTI infections because of additional virulence factors associated with bacteriocin encoding determinants. Although the first explanation may also apply to the higher incidence of colicin E1 plasmids in the UTI, it is unlikely that there are any additional virulence determinants on pColE1 plasmids besides the colicin E1 determinant itself. The size of previously published ColE1 plasmids varied from 5.2 kb [14] to 9 kb in the *E. fergusonii *EF3 strain [38] and contained regions important for plasmid replication, mobilization, and for colicin synthesis. No known virulence determinants have been identified on these plasmids. As shown previously, colicin E1 can kill both normal and cancer eukaryotic cells and this effect has been shown to be cell-specific [39,40]. The toxic effect of colicin E1 on uroepithelial cells could be one of the potential virulence mechanisms found in UPEC strains.

When compared to controls, producer strains with the combination of colicins Ia, E1, and mV were more common in the UTI group. As shown by Jeziorowski and Gordon [28], when colicin Ia and microcin V occur together, they are encoded on the same conjugative plasmid as a result of integration of the microcin V operon and several other genes into the pColIa plasmid. Therefore we tested whether similar integration of colicin E1 genes into the pColIa could explain the observed association of colicin E1 and colicin Ia synthesis. Among the 12 randomly picked colicin E1-synthesizing multi-producers, all strains contained pColE1 DNA that was not recognized by the probe complementary to the colicin Ia-encoding DNA and *vice versa*, suggesting that pColE1 was independently co-associated with pColIa in UTI strains. Moreover, pColE1 sizes were similar to those published previously (5.2 kb, [14]; 9 kb, [38]) indicating that the pColE1 DNA is unlikely to encode any known virulence factor. This finding suggests that colicin E1 itself is a potential virulence factor of certain uropathogenic strains of *E. coli*. However, it is possible that strains carrying colicin E1 genes differ in their genetic content and contain elements promoting their urovirulence. Since it is known that colicin E1 is independently associated with *E. coli *phylogroups [26], the first explanation appears more probable.

## Conclusions

*E. coli *strains isolated from human urinary tract infections showed increased incidence of microcin H47 and colicin E1 production, respectively, and belonged more often to phylogroup B2 when compared to control *E. coli *strains. In the UTI group, producers of 3 or more identified bacteriocin types were more common. Moreover, the incidence of colicin E1 production was high in the UTI multi-producer bacteriocinogenic strains. Unlike colicin Ia- and microcin V-encoding determinants [28], pColE1 was independently associated with pColIa in the UTI strains. Thus, colicin E1 itself appears to be a potentially important virulence factor of certain uropathogenic strains of *E. coli*.

## Methods

### Bacterial strains

Altogether, 772 human *E. coli *strains were isolated between May 2007 and June 2009, from both male and female patients. Five hundred and fifty-nine strains were collected from the Faculty Hospital Bohunice, Brno, CZ, including 361 *E. coli *strains isolated from urinary tract infections (UTI) and 198 *E. coli *strains isolated from feces of patients without bacterial gut infections (control commensal strains). Additional 213 strains of *E. coli *(isolated from feces of patients without bacterial gut infections) were collected from the St. Ann's Faculty Hospital, Brno, CZ. Out of 411 *E. coli *control strains (190 of male and 221 of female origin), only 92 (22.4%) stemmed from patients with primary diagnoses related to the gastrointestinal system (e.g. pancreatitis, dyspepsia etc.) and none were isolated from cases with detectable bacterial intestinal infection. Since no statistically significant differences in the incidence of producer strains or the incidence of individual bacteriocin types between control groups from both hospitals were found, strains from both groups were merged and treated as a single group. UTI strains were isolated from 85 males and 276 females. Bacterial identification of *E. coli *was performed using a set of biochemical reactions (ENTEROtest 16, PLIVA-Lachema Diagnostika, Czech Republic). All donors of investigated strains were Caucasians living in the South Moravia region of the Czech Republic. For each sample, the primary diagnosis of the source patient was established by an experienced clinician.

A described set of *E. coli *indicator strains was used to identify the colicin and microcin types produced: *E. coli *K12-Row, C6 (ϕ), B1, P400, and *Shigella sonnei *17 [1]; additionally, one recently verified indicator strain, *E. coli *S40, was also used [41]. Together, these indicator strains are capable of detecting all known colicin types including colicin L (P400) and colicin Js (*S.s*. 17).

Control bacterial producers encoding different colicin types were taken from laboratory stock and comprised *E. coli *BZB2101pColA - CA31, BZB2102 pColB - K260, BZB2103 pColD - CA23, BZB2107 pColE4 - CT9, BZB2108 pColE5 - 099, BZB2150 pColE6 - CT14, BZB2120 pColE7 - K317, BZB2279 pColIa - CA53, BZB2202 ColIb - P9, BZB2116 pColK - K235, PAP1 pColM - BZBNC22, BZB2123 pColN - 284 (original source: A. P. Pugsley), *E. coli *189BM pColE2 - P9 (B. A. D. Stocker), *E. coli *385/80 pColE1, pColV (H. Lhotová), *E. coli *185M4 pColE3 - CA38 (P. Fredericq), *E. coli *W3110 pColE8, W3110 pColE9 (J. R. James), *E. coli *K-12 pColS4 (D. Šmajs), *S. boydii *M592 (serovar 8) pColU (V. Horák), *E. coli *K339 pColY (D. Friedman), *Shigella sonnei *(colicinotype 7) pColJs (J. Šmarda), *E. coli *pCol5 and *E. coli *pCol10 (H. Pilsl). As microcin control producers, the following bacterial strains were used: *E. coli *449/82 pColX (microcin B17); *E. coli *313/66 pColG (microcin H47); *E. coli *363/79 pColV (microcin V, original source: H. Lhotová); *E. coli *TOP10F' pDS601 (microcin C7); *E. coli *D55/1 (microcin J25); *E. coli *B1239 (microcin L, D. Šmajs).

### Cultivation conditions

The ability to produce bacteriocins of all the strains was tested in parallel on 4 different agar plates containing (i) TY medium, (ii) nutrient broth, (iii) TY medium supplemented with mitomycin C, and (iv) TY medium supplemented with trypsin. The rich TY medium consisted of yeast extract (Hi-Media, Mumbai, India) 5 gl^-1^, tryptone (Hi-Media) 8 gl^-1^, sodium chloride 5 gl^-1^; the TY agar consisted of a base layer (1.5%, w/v, solid agar) and a top layer (0.7%, w/v, soft agar). As a relatively unenriched medium, a Difco™nutrient broth (Difco Laboratories, Sparks, MD) 8 gl^-1^, NaCl 5 gl^-1^, was used for 1.5% (w/v) agar plates. For induction of colicin production, the base agar layer was supplemented with 0.01% (w/v) mitomycin C. To test protease sensitivity of the inhibitive agents, 0.1% (w/v) trypsin was added to the base layer of agar.

### Detection of colicin producers

The agar plates were inoculated by needle stab with fresh broth cultures and the plates were incubated at 37°C for 48 hours. The bacteria were then killed using chloroform vapors and each plate was then overlaid with a thin layer of soft agar containing 10^7 ^cells ml^-1 ^of an indicator strain. The plates were then incubated at 37°C overnight. All 772 *E. coli *strains of clinical origin were tested on four parallel plates against all 6 indicators, i.e. each strain underwent 24 individual tests.

### Identification of colicin and microcin types and determination of *E. coli *phylogenetic group

Identification of individual colicin types (colicins A, B, D, E1-E9, Ia, Ib, Js, K, M, N, S4, U, Y, 5 and 10) was performed using PCR with primers designed using the Primer3 program [42] or with previously published primers [26]. The list of primer pairs and the corresponding length of PCR products are listed in Additional file 1. Total bacterial DNA was isolated using DNAzol (Invitrogen, Carlsbad, CA) reagent according to the manufacturer's protocol. After 100-fold dilution, this DNA was used as a template for PCR reactions. Alternatively, all producer strains were tested with colony PCR. A bacterial colony was picked with a sterile inoculation loop and resuspended in 100 μl of autoclaved water. For each individual PCR reaction, 1 μl of cell suspension was added to the reaction. The PCR detection protocol was as follows: 94°C (2 minutes); 94°C (30 seconds), 60°C (30 seconds), 72°C (1 minute), 30 cycles; 72°C (7 minutes). For DNA amplification directly performed from lysed whole cells (colony PCR), the initial step was extended to 5 minutes (94°C, 5 minutes). All primers detecting colicins were tested against all type producers to reveal potential cross-reactivity of sequentially similar colicin genes. The primer sequences for PCR detection of microcins B17, H47, J25, L, and V, respectively, were taken from previously published paper [26]. With the exception of microcin M, all bacteriocin genes detected in the study performed by Gordon and O'Brien [26] were analyzed in this work. Moreover, 12 additional bacteriocin genes were detected by us. PCR products resulting from detection of sequentially related colicin genes (colicins E2-E9, Ia-Ib, U-Y, and 5-10, respectively) were subjected to dideoxyterminator sequencing using amplification primers. Because of sensitivity of microcin H47 to chloroform vapours, all investigated strains were tested for the presence of microcin H47-encoding genes. Sequence analysis was performed using Lasergene software (DNASTAR, Inc., Madison, WI). The phylogenetic group of each *E. coli *strain was determined using the triplex PCR protocol described previously [27].

### Statistical analyses

Statistical significance of the incidence of genotypes and colicin or microcin types, in both strain groups, was performed by applying standard methods derived from the binomial distribution, including the two-tailed test. *STATISTICA *version 8.0 (StatSoft, Tulsa, OK, USA) was used for statistical calculations. Alternatively, an interactive calculation tool for chi-square tests of "goodness of fit" and independence was used for the calculation of statistical significance of obtained results [43].

### Southern blot analyses and XL-PCR

The total plasmid DNA of selected colicin producers were isolated using QIAprep Spin Miniprep Kit and QIAGEN Plasmid Midi Kit (Qiagen, Hilden, Germany), respectively. During isolation of plasmid DNA, manufacturer's recommendations were followed. The plasmid DNA was digested with the *Eco*RI restriction endonuclease (New England Biolabs, Ipswitch, MA) and the undigested and digested total plasmid DNA was transferred to the Hybond-XL membrane by a standard capillary method (Amersham, Buckinghamshire, UK). The colicin E1 and Ia probes used in Southern blot analysis were amplified from the control producer strains with primers used for detection of colicin genes (Additional file 1). The probes were labelled with the Gene Images AlkPhos Direct Labelling and Detection System (Amersham) and the labelled hybridized probes were detected with the ECF chemifluorescent substrate and the Typhoon imager (Amersham) according to the manufacturer's recommendations.

The GeneAmp^® ^XL PCR Kit (Roche Molecular Systems, Branchburg, NJ, USA) was used for amplification of pColE1 plasmid DNA using pColE1-seq1 (5' - GCCGATCGTGATGCTATTTT - 3') and pColE1-seq2 (5' - AAAATAGCATCACGATCGGC - 3') complementary primers recognizing colicin E1 operon.

## Authors' contributions

DS designed the study and wrote the manuscript. LM and JS performed bacteriocin testing of *E. coli *strains and analyzed the obtained data. MV, AS, ZV and VW contributed to isolations and characterizations of the bacterial strains and gathered data. All authors read and approved the final manuscript.

## Supplementary Material

Additional file 1 **DNA Primers used for PCR detection of colicin and microcin encoding genes**. Click here for file
